# Effect of caffeine on SPECT myocardial perfusion imaging during regadenoson pharmacologic stress: a prospective, randomized, multicenter study

**DOI:** 10.1007/s10554-014-0419-7

**Published:** 2014-04-17

**Authors:** Furqan H. Tejani, Randall C. Thompson, Rita Kristy, Stan Bukofzer

**Affiliations:** 1 Long Island College Hospital, 339 Hicks Street, Brooklyn, NY 11201 USA; 2 St Luke’s Mid America Heart Institute, 4330 Wornall Road, Kansas City, MO 64111 USA; 3 Astellas Pharma Global Development, Inc., 1 Astellas Way, Northbrook, IL 60062 USA; 4 Lake Forest, IL USA

**Keywords:** Caffeine, Myocardial perfusion imaging, Pharmacologic stress, Regadenoson

## Abstract

A multicenter, double-blind, randomized study was conducted to assess the effect of caffeine on regadenoson stress myocardial perfusion imaging (MPI). Subjects with a high likelihood of coronary artery disease underwent a rest single-photon emission computed tomography MPI on day 1 (MPI-1) and a stress MPI with regadenoson on day 3 (MPI-2). Individuals with ≥1 segment with a reversible defect received double-blind caffeine tablets (200 or 400 mg) or placebo 90 min before a repeat regadenoson stress MPI (MPI-3) on day 5. Overall, 207 subjects completed the study (caffeine 200 mg, *n* = 70; caffeine 400 mg, *n* = 71; placebo, *n* = 66). The mean number of segments with reversible defects decreased from MPI-2 to MPI-3 in the caffeine 200 and 400 mg groups versus no significant change in the placebo group [mean ± standard deviation: −0.61 ± 1.097, −0.62 ± 1.367, and 0.12 ± 0.981, respectively (overall treatment effect, *P* < 0.001)]. The majority of subjects who received caffeine shifted to a lower ischemia size category from MPI-2 to MPI-3, with no clear pattern observed in subjects who received placebo. For caffeine exposed patients with ≥3 segments with reversible defects at MPI-2, 21/23 had fewer detected at MPI-3. Both the 200 and 400 mg doses of caffeine significantly reduced the number of segments with reversible defects detected by regadenoson stress MPI.

## Introduction

Regadenoson was approved by the United States Food and Drug Administration in 2008 as a pharmacologic stress agent for myocardial perfusion imaging (MPI) in subjects unable to undergo adequate exercise stress [1]. The special features of this adenosine A_2A_ receptor agonist have been reviewed previously [2]. Additionally, Phase 4 trials have assessed the safety and tolerability of regadenoson in patients with chronic kidney disease and in those with asthma or chronic obstructive pulmonary disease [3, 4].

Caffeine is a nonspecific competitive antagonist of all adenosine receptor subtypes [5, 6]. Therefore, caffeine may interfere with the coronary vasodilatory effects of adenosine agonists through its effects on A_2A_ receptors, which mediate coronary vasodilation [7]. A large majority of the adult population consumes caffeine daily, and the possible interaction of caffeine on the accuracy of stress testing with agents such as regadenoson is an important clinical issue. Regadenoson prescribing information [1] and current American Society of Nuclear Cardiology (ASNC) imaging guidelines [8] both recommend that caffeine and other methylxanthine-containing compounds be withheld for 12 h before vasodilator stress MPI. A previous study showed that moderate oral caffeine intake (200 mg) did not significantly affect regadenoson-induced myocardial blood flow as assessed by positron emission tomography (PET) in 41 healthy individuals [9]. However, that study did not evaluate the effect on the detection of reversible perfusion defects by single-photon emission computed tomography (SPECT).

The objective of the current study was to determine whether the prior administration of caffeine affects the diagnostic accuracy of regadenoson-stress SPECT MPI for detecting reversible defects in subjects with a high likelihood of coronary artery disease (CAD). The safety and tolerability of concomitant caffeine and regadenoson administration were also assessed. A detailed discussion of the rationale and description of the study design has been published previously [10].

## Methods

This Phase 3b, double-blind, randomized, placebo-controlled, parallel-group study (NCT00826280) was conducted at 24 sites in the United States from 24 March 2009 (first subject enrolled) to 15 July 2010 (last evaluation).

### Participant selection

The study involved clinically stable male and female outpatients ≥18 years of age, with a high likelihood of having ischemia on testing, but with an intermediate or low risk of needing immediate coronary intervention. The subjects were also regular caffeine consumers (≥1 cup of caffeinated coffee per day or equivalent). Key inclusion and exclusion criteria have been detailed previously [10].

### Study design

Subjects were screened during a first clinic visit. MPI was conducted on days 1, 3, and 5 as follows: (1) day 1 (baseline): rest MPI (MPI-1); (2) day 3: stress MPI with open-label regadenoson 400 µg (MPI-2). Subjects with ≥1 segment with a reversible defect, as read by the site, (if the apex was involved, then an additional reversible segment had to be present) continued to MPI-3; (3) day 5: stress MPI (MPI-3) in a double-blind, randomized sequence (1:1:1) with regadenoson + placebo, regadenoson + caffeine 200 mg, or regadenoson + caffeine 400 mg. The caffeine intake was approximately equivalent to 2–4 cups of coffee (moderately high). If a subject was subsequently determined not to have ≥1 segment with a reversible defect according to the core imaging laboratory reading, the subject was discontinued. Regadenoson was administered 90 min after placebo or caffeine intake. A follow-up safety visit was scheduled for 24 h after study drug administration at MPI-3 to identify any unforeseen side-effects. Subsequent clinical management was at the discretion of the investigator and based only on the results of the MPI-1 (rest) and MPI-2 (stress) scans.

### Imaging protocol

Regadenoson 400 µg was administered as a 10-s intravenous 5 mL injection followed by a 5 mL saline flush. The radiotracer (10–15 mCi of technetium-99 m sestamibi or tetrofosmin) was administered 10–20 s after regadenoson. SPECT MPI acquisition was started 60–90 min later using standard protocols as described in the ASNC guidelines [8]. The same radiotracer was used across all three MPI procedures for each subject.

Theophylline or theophylline-containing medications were withheld for 7 days before randomization and for the study duration. Caffeinated foods and beverages, dipyridamole, and calcium channel blockers were prohibited for 24 h and nitrates for 12 h before any visit.

The study was conducted in compliance with the principles of the International Conference on Harmonization of Technical Requirements for Registration of Pharmaceuticals for Human Use and Good Clinical Practice. The protocol was approved by the Institutional Review Board/Independent Ethics Committee of each study site. Each subject provided written informed consent prior to any study-related procedures.

### Imaging analysis

All MPI images for subjects completing the study were interpreted by three independent, blinded readers at a central core imaging laboratory. In addition to visual interpretation by the readers, the images were assessed by computerized quantitation using the Emory Cardiac Toolbox™ (Syntermed, Inc., Atlanta, GA, USA). The number of reversible ischemic defects was assessed using a 17-segment model [11]. Segments were considered to have a reversible defect if the stress perfusion score exceeded the rest score and the stress score was ≥2. Scores were based on tracer activity in each segment on a 5-point scale for radiotracer uptake: 0 = normal uptake; 1 = slightly reduced uptake; 2 = moderately reduced uptake; 3 = severely reduced uptake; and 4 = absent uptake. Total scores across all 17 segments were generated as follows: the summed stress score (SSS); the summed rest score (SRS); and the summed difference score (SDS), calculated as the difference between the SSS and SRS. Independent review of SPECT MPI data was facilitated by a configuration of two compliant computerized systems working in tandem: (1) the image review software (Emory Cardiac Toolbox™; Syntermed, Atlanta, GA, USA) and (2) the data capture system incorporating the electronic case report form.

The primary efficacy variable was the change in number of segments with reversible defects between MPI-2 (regadenoson alone) and MPI-3 (regadenoson + placebo or caffeine). Secondary efficacy variables included the change in SDS between MPI-2 and MPI-3, and agreement rates between MPI-2 and MPI-3 according to ischemia size category (0 to <2, 2 to <5, and ≥5 segments with reversible defects, consistent with the categorization applied in the Phase 3 regadenoson trials) [12, 13].

### Safety assessments

Safety was assessed throughout the trial, based on vital signs, adverse events, laboratory assessments, blood cardiac markers (CPK-MB, CPK-MB fraction, and troponin T), and 12-lead electrocardiographs.

### Statistical analyses and sample size calculation

An analysis of variance (ANOVA) and Fisher’s least significant difference with a type 1 error rate of 5 % was used to test for significantly different mean changes in the number of segments with reversible defects among the treatment arms. This approach was able to detect a change of ±1 reversible defect relative to placebo with 95 % power for 200 subjects randomized 1:1:1 with a standard deviation (SD) similar to that observed in the Phase 3 trials (SD = 1.59), and with placebo having a mean effect of 0 on the change in number of segments with reversible defects.

The full analysis set comprised all randomized subjects with 3 interpretable MPI scans, the per-protocol set comprised a subset of full analysis subjects with no major protocol deviations, and the safety analysis set comprised all subjects who received ≥1 dose of regadenoson. Subsets of patients with ≥3 segments with reversible defects at MPI-2 and with SDS scores ≥2 were also examined, to quantify results in patients with a greater disease burden. The two caffeine dose groups were combined for subset analyses.

For the primary efficacy variable, an analysis of covariance (ANCOVA) was used with treatment arm as a factor and the number of segments with reversible defects at MPI-2 as a covariate. Fisher’s least significant difference was used to test for an overall treatment arm effect using a 5 % type 1 error rate.

Additional analyses were conducted on the full analysis set to determine agreement between MPI-2 and MPI-3 with respect to ischemia size category using the median count across the three independent readers. The agreement rate and a 95 % Clopper–Pearson confidence interval (CI) was calculated for each ischemia size category within each treatment arm. The difference in agreement rate between the treatment arms and associated 95 % CI was also calculated for each ischemia size category. A test of marginal homogeneity was performed for each treatment arm. A Cochran–Mantel–Haenszel test of equality of mean scores between MPI-2 and MPI-3 was performed using the following scoring system: 0 to <2 = 0; 2 to <5 = 1; and ≥5 = 2.

The variability of the change in number of segments with reversible defects for the caffeine 200 and 400 mg arms was compared with the placebo arm. Levene’s test was performed with a 5 % type 1 error rate to test for an increase in the variance of each caffeine arm versus placebo if the variance of a caffeine group was greater than the placebo group.

The overall change in SDS was analyzed using the same methods as the primary analysis, with the corresponding variable at the initial stress scan used as a covariate.

To examine if caffeine consumption lowered the rate of headaches, a generalized estimating equation was used due to the correlation between repeated measurements on the same subject. A logistic model was fitted with the dichotomized values of Headache or No headache and to assess the significance of treatment effect.

## Results

### Study population

**Fig. 1:**
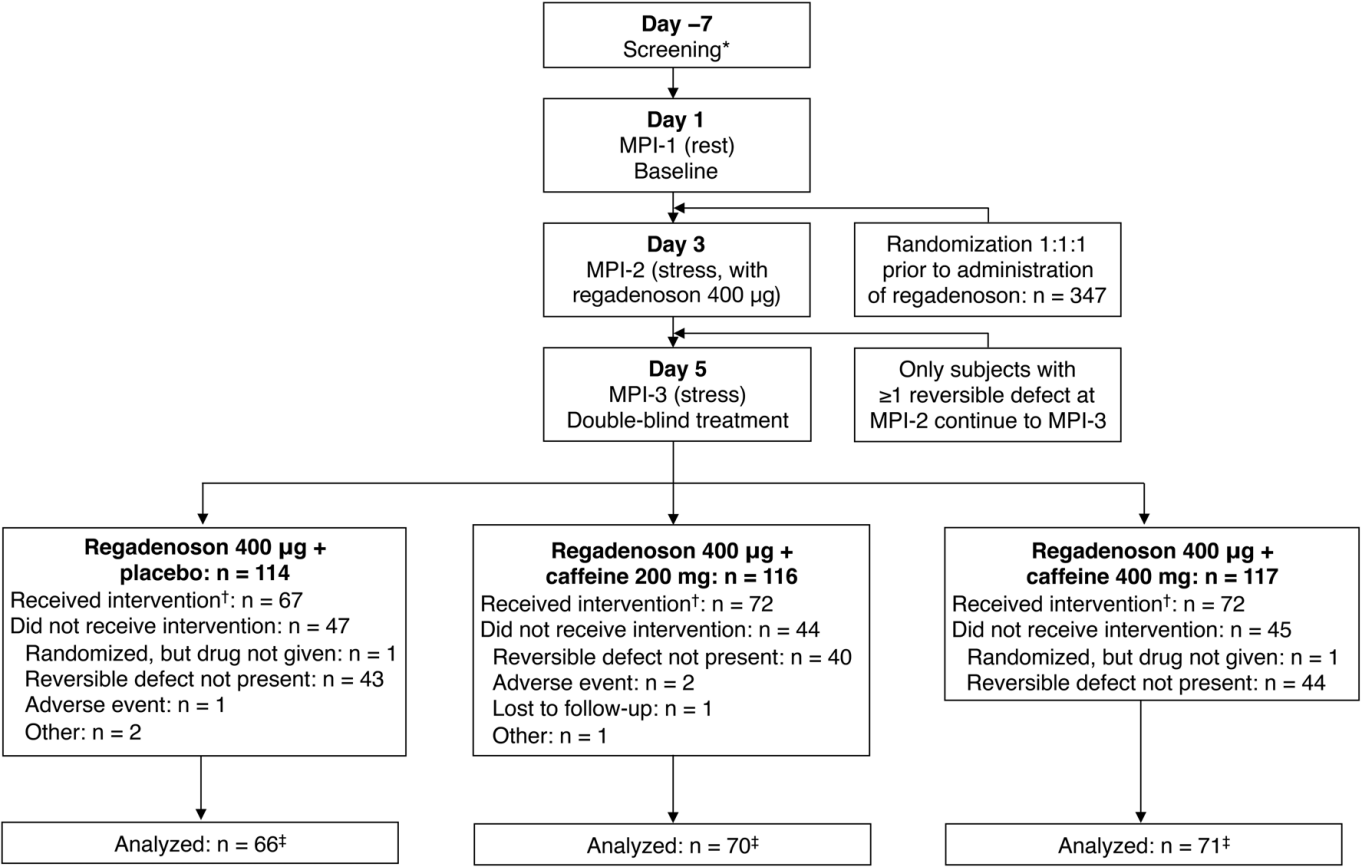
Patient disposition. *Data was not collected on the number of subjects screened, only those randomized. ^†^Received randomized treatment at MPI-3 (regadenoson plus placebo or caffeine). ^‡^All subjects with interpretable MPI-1, MPI-2, and MPI-3 scans

Of the 347 subjects randomized, 345 received at least one dose of study drug (safety analysis set) (Fig. 1). The full analysis set included 207 subjects. The absence of a reversible ischemic defect at MPI-2 was the major reason for study discontinuation. A total of 29 patients had ≥3 segments with reversible defects at MPI-2.

Baseline demographic and clinical characteristics were generally consistent between the regadenoson + placebo and regadenoson + caffeine groups (Table 1). The majority of the study population was comprised of white males.

**Table 1:** Baseline demographics and clinical characteristics (full analysis set)

	Regadenoson + placebo (*n* = 66)	Regadenoson + caffeine 200 mg (*n* = 70)^a^	Regadenoson + caffeine 400 mg (*n* = 71)	*P* value^†^
Sex, *n* (%)				0.1842
Male	55 (83.3)	58 (82.9)	51 (71.8)	
Female	11 (16.7)	12 (17.1)	20 (28.2)	
Ethnicity, *n* (%)				0.8753
Non- Hispanic or Latino	60 (90.9)	64 (91.4)	63 (88.7)	
Hispanic or Latino	6 (9.1)	6 (8.6)	8 (11.3)	
Race, *n* (%)				0.7030
White	61 (92.4)	63 (90.0)	68 (95.8)	
Black/African American	5 (7.6)	5 (7.1)	3 (4.2)	
Other	0	2 (2.9)	0	
Age (years)				0.0858
Mean ± SD	68.0 ± 10.0	65.7 ± 11.1	69.4 ± 8.2	
Range	43–91	32–86	46–86	
Weight, kg, mean ± SD	99.2 ± 22.8	98.4 ± 23.1^b^	94.0 ± 19.4	0.3153
Concomitant medications, *n* (%)				0.6937
Beta-blocking agents	52 (78.8)	59 (84.3)	58 (81.7)	

*SD* standard deviation

^†^To compare for differences across treatment groups, a 1-way analysis of variance was used for continuous variables and Fisher’s exact test (2-tailed) was used for the discrete variables

^a^Unless otherwise stated

^b^ *n* = 69

### Efficacy analyses

**Fig. 2:**
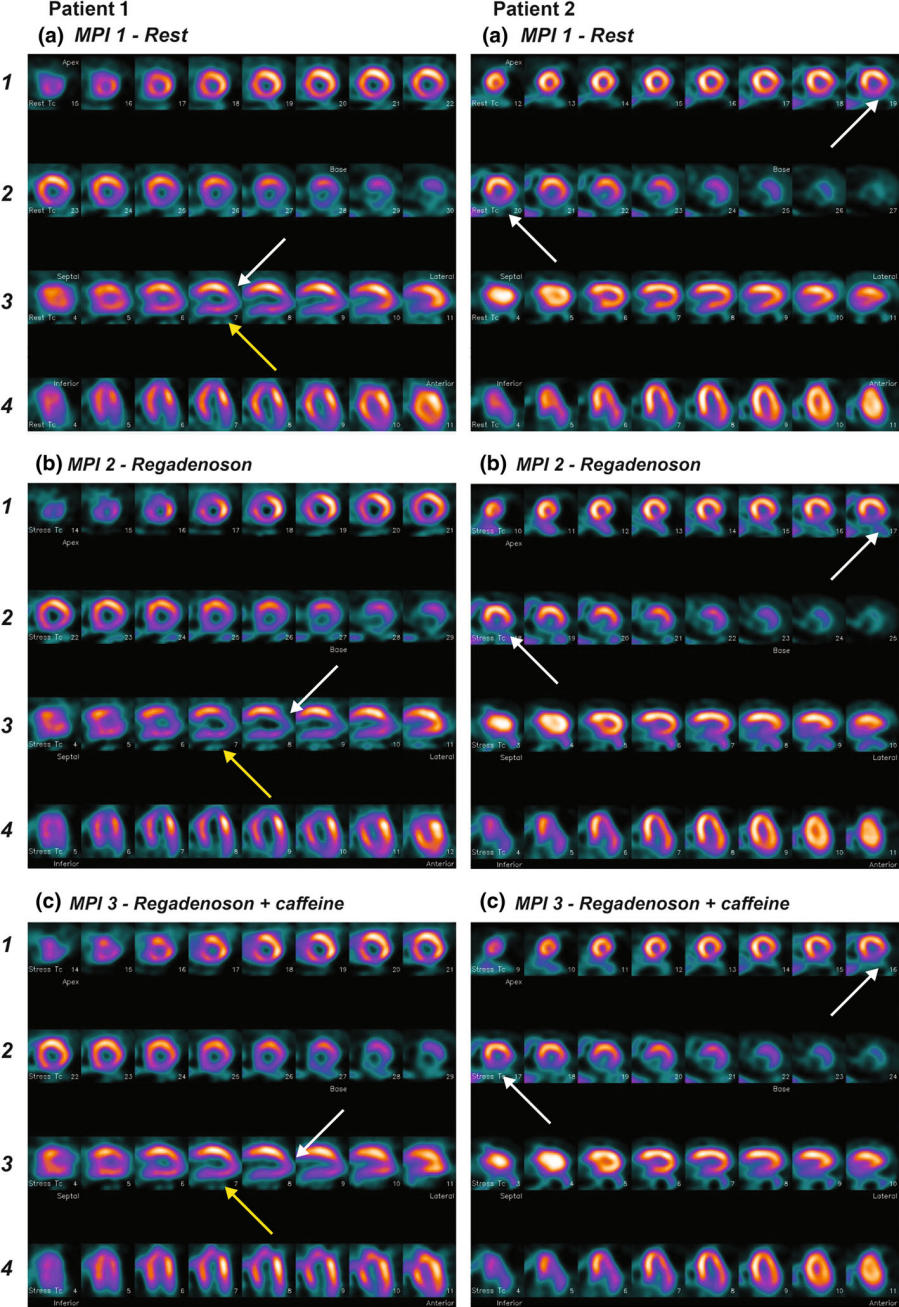
Example images. *Patient 1* images show a predominantly reversible inferior left ventricular defect (*yellow arrows*), and a predominantly reversible defect at the left ventricular apex (*white arrows*). Both defects are more intense after regadenoson stress (**b**) than after regadenoson plus caffeine 200 mg (**c**). There is also a nonreversible defect infero-laterally. *Patient 2* images show a partially reversible inferior left ventricular defect (*white arrows*). The defect is larger and more intense after regadenoson stress (**b**) than after regadenoson plus caffeine 200 mg (**c**)

Example images are shown in Fig. 2. The mean (±SD) number of segments with reversible defects based on blinded reader assessment did not change significantly from MPI-2 to MPI-3 in the placebo group (0.12 ± 0.981; *P* = 0.3192), but decreased in the caffeine 200 mg (−0.61 ± 1.097; *P* < 0.001 vs. placebo) and 400 mg (−0.62 ± 1.367; *P* < 0.001 vs. placebo) groups (*P* < 0.001 for the overall treatment effect; Table 2). The mean change using automated quantitation was consistent with the results from the blinded reader analysis (Table 2). There was no significant difference between the two caffeine dose groups (*P* = 0.9328; Table 2).

**Table 2:** Mean number and mean change in number of segments with reversible defects between MPI-2 and MPI-3, as assessed by blinded readers and computerized quantitation (full analysis set)

	Regadenoson + placebo, *n* = 66	Regadenoson + caffeine 200 mg (*n* = 70)	Regadenoson + caffeine 400 mg (*n* = 71)	Overall treatment effect *P* value*
Blinded reader analysis				
Number of segments with reversible defects, mean ± SD				
MPI-2 (regadenoson alone)	0.67 ± 1.377	1.01 ± 1.452	1.00 ± 1.595	
MPI-3 (regadenoson + placebo or caffeine)	0.80 ± 1.511	0.40 ± 0.907	0.38 ± 0.962	
Change in number of segments with reversible defects, mean ± SD	0.12 ± 0.981	−0.61 ± 1.097	−0.62 ± 1.367	<0.001
*P* value versus placebo^†^		< 0.001	< 0.001	
*P* value versus caffeine 200 mg^†^			0.9328	
Computerized quantitation analysis				
Number of segments with reversible defects, mean ± SD				
MPI-2 (regadenoson alone)	1.47 ± 1.927^a^	2.00 ± 2.364^b^	2.19 ± 2.122^c^	
MPI-3 (regadenoson + placebo or caffeine)	1.74 ± 2.355	1.46 ± 1.954	1.42 ± 1.794	
Change in number of segments with reversible defects, mean ± SD	0.31 ± 1.622^a^	−0.59 ± 1.743^b^	−0.81 ± 1.812^c^	0.0037
*P* value versus placebo^†^		0.0089	0.0016	
*P* value versus caffeine 200 mg^†^			0.5654	

*SD* standard deviation

* *P* value is from the primary analysis using analysis of covariance

^†^The unadjusted *P* values for pairwise differences should be used for interpretation only if the *P* value for the treatment effect is ≤0.05

^a^n = 64

^b^n = 69

^c^n = 70

**Fig. 3:**
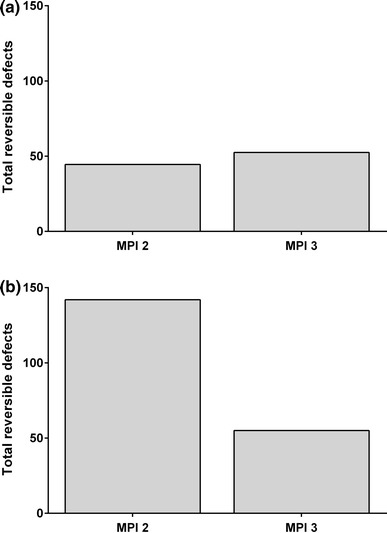
Number of segments with reversible defects detected during MPI 2 and MPI 3 in patients who received placebo (**a**) and caffeine (**b**)

**Fig. 4:**
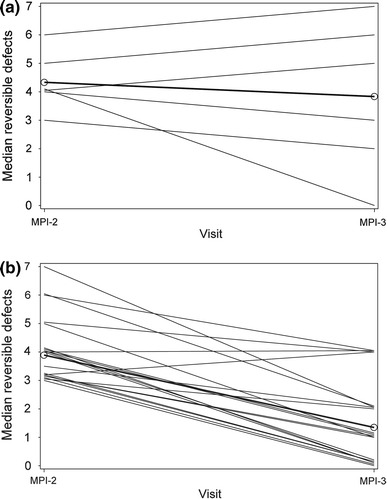
Median number of segments with reversible defects detected at MPI-2 and MPI-3 in subjects with ≥3 segments with reversible defects at MPI-2 who received placebo (**a**) or caffeine 200 or 400 mg (**b**) at MPI-3

The total number of segments with reversible defects detected (the sum of the median response for the three blinded readers) was 2.6-fold lower from MPI-2 to MPI-3 in the combined 200 and 400 mg caffeine groups, whereas there was little difference from MPI-2 to MPI-3 in the placebo group (Fig. 3). In the subset of patients with ≥3 segments with reversible defects at MPI-2, the median number of segments with reversible defects at MPI-2 and MPI-3 indicated that fewer reversible defects were detected following caffeine (200 and 400 mg groups combined), whereas there was no such pattern in the placebo group (Fig. 4). Of these caffeine exposed patients, 21/23 with ≥3 segments with reversible defects at MPI-2 had fewer defects detected at MPI-3. Of the subjects who received caffeine, 26/36 (72 %) of subjects with 2 to <5 segments with reversible defects and 1/5 (20 %) of subjects with ≥5 segments with reversible defects at MPI-2 shifted to the non-ischemic category (0–1 defects) at MPI-3. Therefore, a total of 27/41 (66 %) shifted from ischemic to non-ischemic and 3/100 (3 %) subjects shifted from non-ischemic to ischemic. In the placebo group, 4/14 (29 %) subjects shifted from ischemic to non-ischemic, and 5/52 (10 %) shifted from non-ischemic to ischemic.

**Fig. 5:**
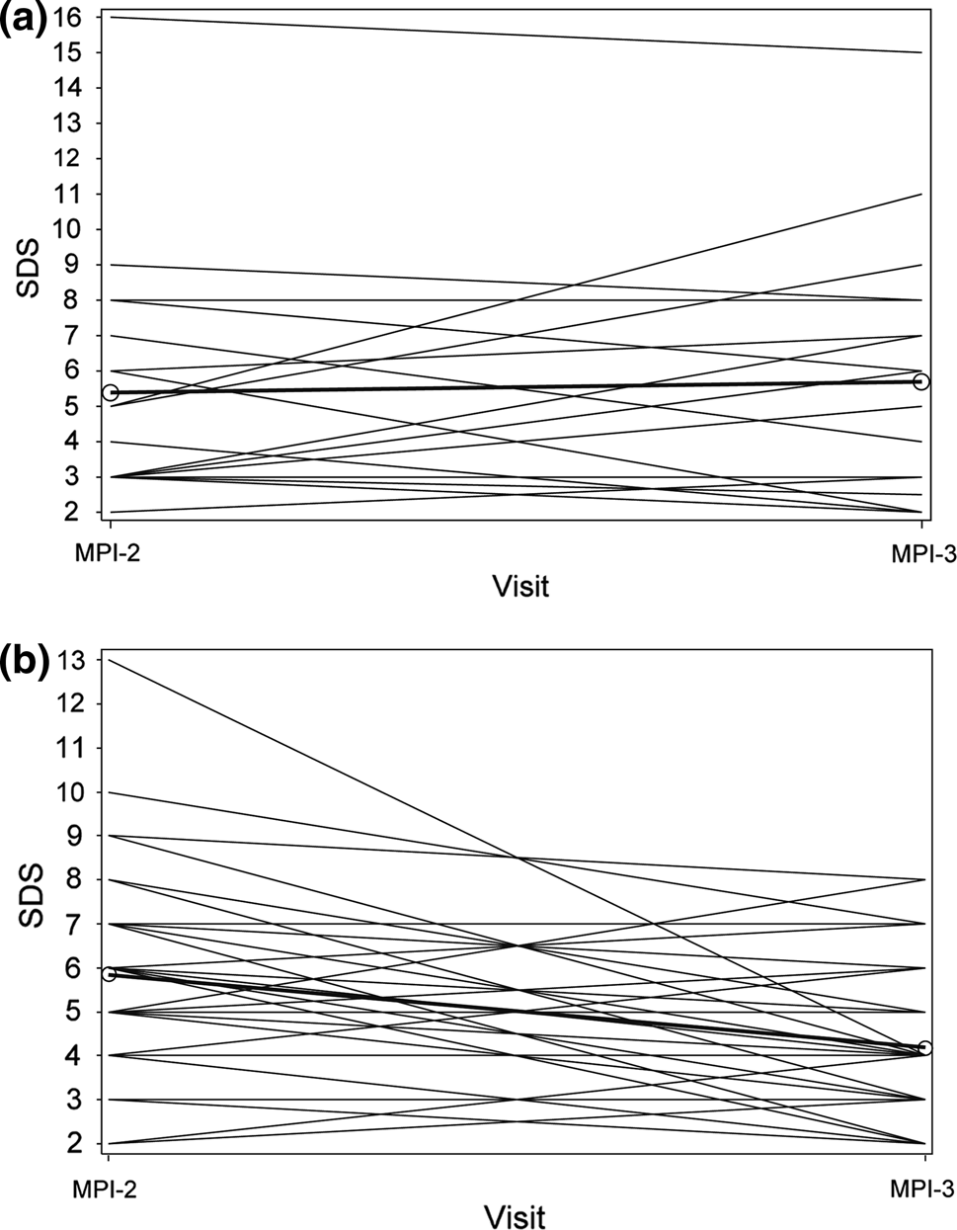
Summed difference scores (SDS) at MPI-2 and MPI-3 in subjects with a SDS score ≥2 who received placebo (**a**) or caffeine 200 or 400 mg (**b**) at MPI-3

The mean (±SD) change in SDS from MPI-2 to MPI-3, as assessed by the blinded readers, increased slightly in the placebo group (0.11 ± 2.871), but decreased in the caffeine 200 mg (−1.03 ± 2.071; *P* = 0.0034 vs. placebo) and 400 mg (−1.25 ± 2.664; *P* < 0.001 vs. placebo) groups (*P* = 0.0011 for the overall treatment effect; *P* = 0.5902 for comparison of the two caffeine doses). In the subset of patients with an SDS response ≥2 (*n* = 100), a general trend towards a lower score following caffeine (200 and 400 mg groups combined) was observed, whereas there was no such pattern in the placebo group (Fig. 5). Of these caffeine-exposed patients, 31/68 had a lower SDS at MPI-3 compared with MPI-2, whereas 37/68 had an increase or no change in SDS.

Reader agreement rates by the extent of ischemia (0 to <2, 2 to <5, and ≥5 reversible segments) were significantly different between MPI-2 and MPI-3 for both caffeine groups, whereas there was no significant difference in the placebo group (Table 3). For both caffeine groups combined, 26/36 (72 %) of subjects with 2 to <5 reversible segments and 5/5 (100 %) with ≥5 reversible segments at MPI-2 shifted to a lower category at MPI-3 (i.e., less ischemia was detected). There was no clear pattern in the placebo group (Table 3). Overall, agreement rates in the caffeine groups tended to decrease as the ischemia extent increased, being highest for the 0 to <2 category and poorest for the ≥5 category (although sample sizes were small in the latter category).

**Table 3:** Agreement of MPI-2 and MPI-3 with respect to ischemia size category, as assessed by blinded readers (full analysis set)

	Number of segments with reversible defects	MPI-3 (regadenoson + placebo or caffeine)			Agreement rate ± SE	*P* value*
		0 to <2	2 to <5	≥5		
MPI-2 (regadenoson alone)	Placebo (*n* = 66)					0.527
	0 to <2	47	5	0	0.904 ± 0.041	
	2 to <5	4	7	1	0.583 ± 0.142	
	≥5	0	0	2	1.000 ± 0.000	
	Caffeine 200 mg (*n* = 70)					0.000
	0 to <2	48	0	0	1.000 ± 0.000	
	2 to <5	13	7	0	0.350 ± 0.107	
	≥5	1	1	0	0.000 ± 0.000	
	Caffeine 400 mg (*n* = 71)					0.003
	0 to <2	49	3	0	0.942 ± 0.032	
	2 to <5	13	3	0	0.188 ± 0.098	
	≥5	0	3	0	0.000 ± 0.000	

*SE* standard error

* *P* value is for testing equality of MPI-2 and MPI-3 mean scores (0 to <2 = 0; 2 to <5 = 1; ≥5 = 2)

Transient ischemic dilatation (TID) was not coded prospectively by the core lab readers. The extent of ischemia was quite modest in most patients and based on review of the scans with significant ischemia, there does not appear to be any clinically significant TID.

### Safety and tolerability

Based upon the safety analysis set, similar proportions of subjects in the 3 treatment groups reported adverse events (Table 4), the most common being dyspnea and headache. Most adverse events were of mild or moderate intensity. The number of adverse events considered by the investigator as possibly or probably related to regadenoson was also balanced across the three groups. Adverse events led to the discontinuation of one subject in the regadenoson + placebo group (elevated cardiac markers) and three subjects in the regadenoson + caffeine 200 mg group (infusion site extravasation, angina pectoris [serious adverse event, considered unrelated to regadenoson or caffeine by the investigator], and nausea and vomiting). A post hoc analysis in patients who received intervention showed that the chance of having a headache at MPI-3 was 51 % lower for subjects who received caffeine 200 mg and 70 % lower for those who received caffeine 400 mg versus placebo (Table 5).

**Table 4:** Summary of adverse events (safety analysis set)

AE, *n* (%)	Regadenoson + placebo (*n* = 113)	Regadenoson + caffeine 200 mg (*n* = 116)	Regadenoson + caffeine 400 mg (*n* = 116)
AEs	88 (77.9)	92 (79.3)	87 (75.0)
Regadenoson-related AEs^a^	87 (77.0)	91 (78.4)	84 (72.4)
Caffeine-related AEs^a^	6 (5.3)	7 (6.0)	7 (6.0)
Most common AEs^b,c^			
Dyspnea	43 (38.1)	47 (40.5)	34 (29.3)
Headache^d^	36 (31.9)	37 (31.9)	38 (32.8)
Flushing	27 (23.9)	29 (25.0)	27 (23.3)
Chest discomfort	22 (19.5)	25 (21.6)	19 (16.4)
Dizziness	22 (19.5)	25 (21.6)	18 (15.5)
Nausea	13 (11.5)	16 (13.8)	10 (8.6)
Chest pain	8 (7.1)	1 (0.9)	2 (1.7)
Stomach discomfort	7 (6.2)	4 (3.4)	4 (3.4)
Abdominal discomfort	6 (5.3)	3 (2.6)	5 (4.3)
Abdominal pain upper	6 (5.3)	4 (3.4)	5 (4.3)
Dysgeusia	4 (3.5)	11 (9.5)	5 (4.3)
Feeling hot	3 (2.7)	5 (4.3)	6 (5.2)

All subjects who received ≥1 dose of regadenoson, including those who did not receive the second regadenoson-stress scan (MPI-3)

*AE* adverse event

^a^Possibly or probably related

^b^Medical Dictionary for Regulatory Activities (MedDRA) preferred term

^c^Occurring in >5 % of subjects

^d^Data include patients with any exposure to regadenoson. See post hoc analysis of headache in subjects with baseline and randomized exposure

**Table 5:** Results of a post hoc analysis in patients who received both scans

		MPI-3 (regadenoson + placebo or caffeine)					
		Placebo (n = 67)		Regadenoson + caffeine 200 mg (n = 72)		Regadenoson + caffeine 400 mg (n = 72)	
		No headache	Headache	No headache	Headache	No headache	Headache
MPI-2 (regadenoson alone)	No headache	43	10	47	4	46	3
	Headache	4	10	13	8	17	6

**Fig. 6:**
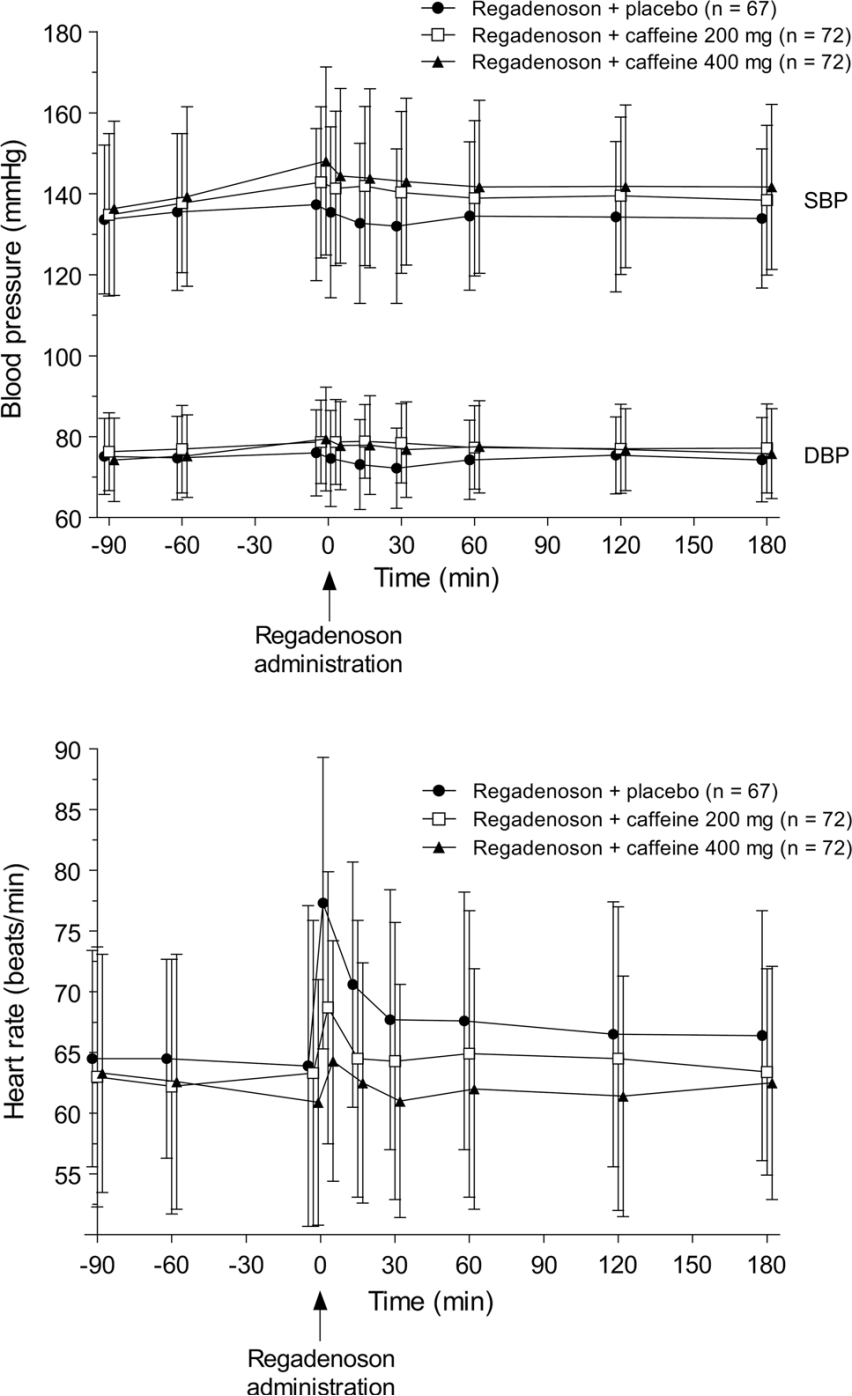
Mean (±SD) systolic blood pressure (SBP), diastolic blood pressure (DBP), and heart rate at MPI-3*. *Blood pressure and heart rate data not available for all subjects at all timepoints

No clinically important differences were noted between the treatment groups in blood cardiac markers, laboratory parameters, physical examination findings, or electrocardiographic abnormalities. Vital sign monitoring at MPI-3 revealed a tendency for systolic blood pressure to be higher after the administration of caffeine versus placebo; the difference (approximately 9 mmHg) was significant (*P* < 0.05) at the 15- and 30-min post-regadenoson assessments for subjects who received 200 mg caffeine, and at all assessments from 3 min before, to 180 min post-, regadenoson for subjects who received 400 mg caffeine (Fig. 6). Diastolic blood pressure was also significantly (*P* < 0.05) increased (by approximately 4–6 mmHg) compared with placebo at the 3-, 15-, and 30-min post-regadenoson assessments in subjects who received 200 mg caffeine, and at the 15- and 30-min post-regadenoson assessments in subjects who received 400 mg caffeine (Fig. 6). The heart rate response to regadenoson appeared to be blunted in subjects who received caffeine, being significantly (*P* < 0.05) lower (by approximately 4–13 bpm) compared with placebo at the 3- and 15-min post-regadenoson assessments in subjects who received 200 mg caffeine, and at all assessments from 3 to 180 min post-regadenoson in subjects who received 400 mg caffeine (Fig. 6).

## Discussion

This is the first prospectively designed, multicenter trial that has been performed to evaluate the interaction between caffeine and a vasodilator stress agent. The primary finding of this study was that caffeine ingestion 90 min prior to regadenoson-stress SPECT MPI reduced the mean number of reversible perfusion defects detected in subjects with a high likelihood of CAD. A general trend towards detection of less ischemia following caffeine was observed, indicating the potential for the diagnostic outcome of regadenoson-stress SPECT MPI being altered by the intake of caffeine prior to the procedure.

Regadenoson is a low-affinity, selective A_2A_ receptor agonist. Because of the large A_2A_ receptor reserve in the coronary arterial bed, low-affinity agonists need only occupy a relatively small proportion of receptors to produce rapid and near-maximal coronary vasodilation [14]. On this basis, it might be expected that caffeine would have a minimal effect on regadenoson-induced coronary hyperemia, as suggested by Gaemperli et al. [9]. These authors found that regadenoson produced an approximate threefold increase in myocardial blood flow in healthy volunteers with and without prior ingestion of caffeine 200 mg. This degree of coronary hyperemia lies well within the level considered adequate to acquire good quality scintigraphic images during stress MPI. Our results, which demonstrate significant attenuation of ischemic defects on SPECT imaging in caffeine-loaded patients are somewhat discordant with those of the study reported by Gaemperli et al. [9]. One would expect that caffeine would either attenuate both regadenoson induced PET coronary flow augmentation and SPECT reversible perfusion defects, or neither. However, there are some important differences between the study reported by Gaemperli et al. and the current study. Firstly, PET myocardial flow calculations take into account a time domain and have their own limitations that are distinct from those of SPECT static relative perfusion images. Secondly, the patients in our study were not normal volunteers but, in accordance with the inclusion criteria, had CAD. Our results are also somewhat discordant with a smaller study by Zoghbi et al. [15]. In that study, adenosine induced SPECT ischemic defects were not attenuated by one 8 oz cup of coffee administered 1 h prior to testing. The average dose of caffeine in our study was higher than in the Zoghbi et al. trial, and the number of patients enrolled was substantially larger (207 compared to 30). Both of these factors might account for the difference in results. It is also possible that caffeine attenuates the coronary dilating effects of regadenoson more than it does adenosine, but since caffeine is a non-specific adenosine antagonist, we believe this explanation is less likely. Since the current study was designed to match clinical practice, and since clinicians use the extent and severity of SPECT reversible defects in decision making, the attenuating effect of caffeine on these defects should guide testing protocols.

The present clinical recommendation for stress MPI with regadenoson, according to the prescribing information and the ASNC imaging guidelines, is to refrain from ingesting caffeine-containing foods or beverages for 12 h before the MPI test [1, 8]. Although this restriction is inconvenient for patients and is often disruptive to the stress laboratory workflow when patients forget to comply, it appears to be necessary. The results of the current study indicate that the inadvertent ingestion of a cup of coffee 90 min prior to the administration of regadenoson could lead to an underestimation of the ischemic burden detected by regadenoson stress MPI. Although the effect of caffeine consumption 12 h before testing was not evaluated, the heterogeneity in caffeine metabolism among individuals and the variability in caffeine content of a cup of coffee suggests that there is a potential for an interaction from caffeine administered several hours prior to regadenoson [16]. The current recommendation to withhold caffeine-containing products for ≥12 h therefore remains appropriate.

The adverse event profile of regadenoson was consistent with previous studies [12, 13, 17]; no unexpected adverse events were reported. In a post hoc analysis, prior intake of caffeine was associated with a decreased incidence of headache at MPI-3 compared with placebo intake in those patients who completed the study. Possible explanations for this finding include blunting of the incidence of caffeine withdrawal headache in the two caffeine groups, and counteraction of regadenoson-induced headache by caffeine. Cessation of caffeine intake among habitual users is associated with a withdrawal headache, which typically develops within 24 h after the previous caffeine ingestion and is alleviated within 1 h of consuming 100 mg caffeine [18, 19].

A number of limitations should be considered regarding the current study results. First, most subjects had only one segment with a reversible defect at MPI-2, with only 14 % of subjects having ≥3 segments with reversible defects. Although this trial was designed to recruit patients with significant ischemia on MPI testing, such recruitment proved to be challenging. This is consistent with the decreasing frequency and severity of abnormal stress SPECT MPI studies since 1991 recently reported by Rozanski et al. [20]. A second limitation is that as subjects did not undergo coronary angiography, the stress scan that most accurately reflected the result is unknown. Lastly, because of considerable variability among individual readings, conclusions based on the mean results should be interpreted with caution.

In conclusion, this study suggests that the consumption of caffeine equivalent to 2–4 cups of coffee 90 min prior to regadenoson-stress SPECT MPI has the potential to adversely affect clinical interpretation of the acquired images and may affect the diagnostic conclusions drawn. These results support pre-procedural directions on caffeine intake as specified in the regadenoson prescribing information and patients should continue to be instructed to avoid consuming caffeine-containing products for ≥12 h prior to the scheduled test.
